# High long non-coding RNA NORAD expression predicts poor prognosis and promotes breast cancer progression by regulating TGF-β pathway

**DOI:** 10.1186/s12935-019-0781-6

**Published:** 2019-03-20

**Authors:** Ke Zhou, Qin Ou, Geng Wang, Wenqi Zhang, Yin Hao, Wenfang Li

**Affiliations:** 1 Department of General Surgery, The Taihe Hospital Affiliated to Hubei University of Medicine, Shiyan, 442000 Hubei China; 2 Department of Pathology, Hubei University of Medicine, Shiyan, 442000 Hubei China

**Keywords:** Breast cancer, LncRNA-NORAD, TGF-β

## Abstract

**Background:**

To investigate the expression and significance of long noncoding RNA NORAD (lncRNA-NORAD) in breast cancer.

**Methods:**

Q-PCR was adopted to detect the mRNA expression of lncRNA-NORAD in breast cancer and adjacent tissues, survival analysis to compare the low-expression groups with the Kaplan–Meier method. Knockout of lncRNA-NORAD was adopted to observe the effects on the cell proliferation, migration and invasion of breast cancer in vitro and in vivo. The TGF-β/RUNX2 signaling pathway was observed by Western blot after the knockout of lncRNA-NORAD.

**Results:**

Increased expression of lncRNA-NORAD in breast cancer tissues promotes proliferation, invasion and migration of breast cancer cells and correlated with worse prognosis. LncRNA-NORAD activated TGF-β/RUNX2 signaling pathway in breast cancer cells.

**Conclusions:**

These results strongly suggested that lncRNA-NORAD might play an important role in breast cancer progression and potentially be a new therapeutic target.

## Background

Breast cancer is the most malignant tumor in women through the world widely, and the incidence of breast cancer is still gradually rising, seriously threatening the women life and health [1]. Although the research on breast cancer has achieved great progress, the pathogenesis of breast cancer remains to be elucidated urgently [2]. Long non-coding RNA (lncRNA), a class of non-coding RNA molecules with about 200 nucleotides, plays an vital role in tumor growth, invasion, metastasis and angiogenesis [3]. So far, many studies have found that lncRNA expression disorders in various cancers, and abnormal lncRNA expression actually occurs in all stages of cancer development [4]. LncRNA could effectively enhance cell growth signal such as Wnt or Akt and thus promote tumor cell proliferation, migration and invasion [5].

It is worth noting that some lncRNAs, including MALAT1, ATB, are involved in TGF-β induced epithelial-mesenchymal transition (EMT) [6, 7]. Recently, studies indicated that lncRNA-NORAD was valuable in the differential diagnosis of colorectal benign and malignant lesions [8]. High lncRNA-NORAD expression promoted EMT transformation and regulated transforming growth factor-β (TGF-β) signaling [9]. High lncRNA-NORAD expression in esophageal cancer was associated with poor prognosis [10]. However, the lncRNA-NORAD expression significance in breast cancer has not be investigated yet.

In the present study, we found that lncRNA-NORAD expression was increased in breast cancer, and that lncRNA-NORAD overexpression promoted the proliferation, migration and invasion of breast cancer cells, and activated the TGF-β signaling pathway. These results strongly suggested that lncRNA-NORAD might play an important role in breast cancer progression and could be a therapeutic target.

## Materials and methods

### Tissue specimens

21 cases of cancer tissue and 10 cases of paracancerous tissue were collected from May 2017 to September 2018 in the Taihe Hospital Affiliated to Hubei Medicine University. Paraffin specimens were removed from 18 cases of breast cancer and 10 cases of hyperplasia of breast tissue.

### RNA extraction

Tissue and cells were collected and cleaved, and an appropriate amount of Trizol solution was added (1 ml Trizol solution was added to the cells of the 6-cm culture plate). The cells were left to fully contact for 1 min, and the cells were transferred to the non-RNA enzyme centrifuge tube. Extraction: 1/5 volume of trichloromethane was added and shaken violently for 1 min. After fully mixing, it was left at room temperature for 3 min. 37 °C, 12,000 RPM centrifuge for 10 min. Transfer the upper RNA layer to the new RNA-free enzyme centrifuge tube. Precipitation: isopropyl alcohol was added, mixed, and left at room temperature for 10 min. 4 °C, 12,000 RPM centrifuge for 10 min. Precipitated RNA could be observed at the bottom. Discard the supernatant and retain the precipitate. Wash: gently add 1 ml of freshly prepared 75% ethanol. Upside-down washing precipitation and pipe wall. 4 °C, 12,000 RPM centrifugal for 5 min. Discard the supernatant and retain the precipitate. Dry at room temperature. Dissolve: add the appropriate amount of DEPC to treat the water and dissolve the RNA. Measure RNA concentration. RNA was detected by agarose gel electrophoresis test.

### Rt-pcr

After completion of RNA extraction, take 1–2 μg RNA, 1 μl Oligo d (T), DEPC water to 8 μl, in 70 °C water bath for 5 min, to move quickly to 4 °C cooling after 5 min. Reaction at the end of each tube in 80**–**180 μl ddH2O (according to the concentration and subsequent experiment needs), and 20 °C saved for later use.

### Western blot

The cells were washed twice in a cold PBS solution and a mixture of protease inhibitors was added to RIPA lysis buffer (50 mM Tris–HCl, pH 7.4, 150 mM NaCl, 1% deoxycholic acid sodium, 1% Triton x-100 and 0.1% SDS). The whole protein was subjected to SDS-PAGE electrophoresis under 30 μg/pore. Transfer the film by wet transfer. Sealing fluid for 5% skimmed milk powder solution containing 0.1% Tween 20, immersed membrane in sealing fluid, 37 °C shaking bed 1 h incubation. Primary antibodies against special proteins diluted with PBST to the appropriate concentration, incubation at 37 °C shake on the bed overnight. PBST was used to wash the membrane 3 times, 5 min each time, and the non-specific binding on the membrane was washed. Then, the membrane was incubated for 40 min with the HRP-labeled second antibody diluted with PBST at 37 °C shaking bed. Wash the film 3 times with PBST for 5 min each. Protein expression was detected by ECL reagent using a chemical image luminescence system.

### Wound healing assay

1 × 10^6^ cells were spread in the 6-well plate. Scratched with 10 μl pipette tip. The migration progress was detected 24 h later and photographed.

### Cell invasion assay

The cells were starved for 24 h, then the 5 × 10^5^ (invasion) cells in serum-free medium were spread to cell compartments with 8 mm holes, and the culture medium containing 10% FBS was added to the pore plate below. For the invasion–determination experiment, the room was coated with Matrigel and stained for 48 h. The cells were stained with crystal violet for 16 h after incubation. Randomly selected areas were photographed and the stained cells were statistically analyzed.

### Tumor xenograft model

The MDA-MB-231 breast cancer cells were transfected with lenti-control (sh-nc) or lenti-shRNA NORAD (sh-NORAD) (GeneChem, Shanghai, China) and selected for puromycin resistance (10 μg/ml). The 4 weeks of BALB/c female mice were bred, and MDA-MB231 cells were cultured. Then, 5 × 10^6^ cells/200 μl PBS/nude mice were injected into the buttocks of the mice, 5 in each of the experimental group and the control group. After the injection of the cells, the tumor’s long diameter and short diameter were observed once a week, and the mice were killed after 6 weeks. The tumor mass was completely removed and photographed. In this part, the calculation formula of tumor volume is L × S/2, L is the tumor’s long diameter and S is the tumor’s short diameter. And the tumor weight was also weighted.

### Statistical analysis

SPSS 17 statistical software was adopted for analysis, t test was used for measurement data, and the Kaplan–Meier analysis was used for survival analysis. *p *< 0.05 was considered statistically significant.

## Results

### High lncRNA-NORAD expression in breast cancer tissues correlated with poor prognosis

**Fig. 1:**
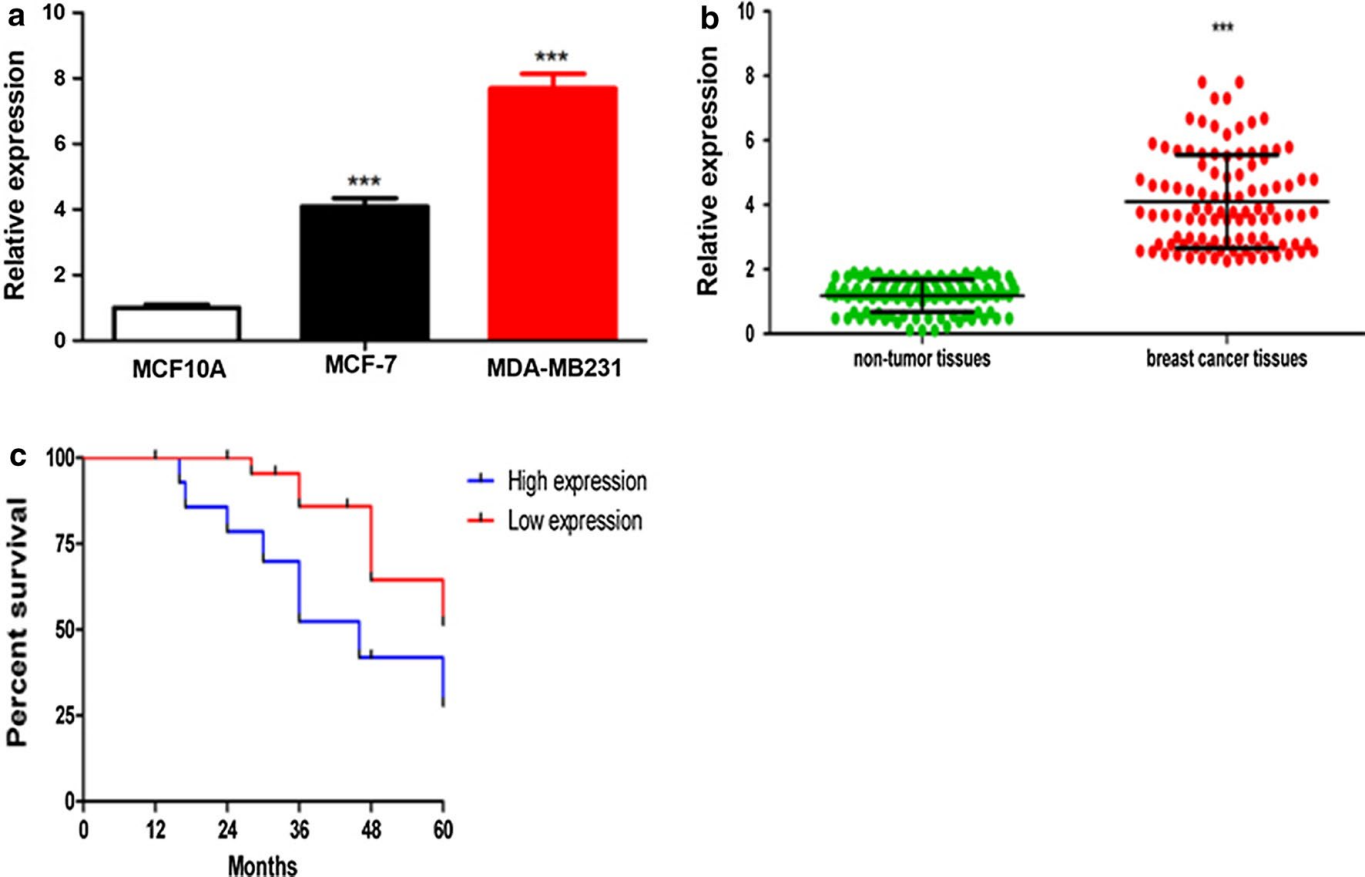
Upregulation of lnc-NORAD in breast cancer cell lines and tumor tissues. **a** Lnc-NORAD RNA levels were detected by RT-PCR in different cell lines. The MCF-7 and MDA-MB-231 cells are breast cancer cells, while the MCF10A cells are normal mammary cells. GAPDH RNA was used as an internal control. Data were averaged from triplicate experiments. **b** Statistical comparison of lnc-NORAD expression levels in patient tissue samples. Lnc-NORAD RNA levels were quantitated from breast tumor tissues and the adjacent normal tissues using the same method described above. ***p < 0.001, vs the control. **c** Survival analysis indicated that the high-expression groups had worse survival compared to the low-expression groups with the Kaplan–Meier method

Aberrant lncRNA-NORAD expression has been found in several cancers, we first detected lncRNA-NORAD expression levels in two breast cancer cell lines (MDA-MB231and MCF-7) and the final results indicated that lncRNA-NORAD were higher than that in the normal mammary cell line (MCF10A) (Fig. 1a). We further detected lncRNA-NORAD in breast cancer tissues and adjacent normal breast tissues, and the results showed that lncRNA-NORAD was higher in breast cancer tissues than normal tissues (Fig. 1b). The whole patients were divided into two groups, the low- and high-expression groups, according to the lncRNA-NORAD levels in the tumor. And survival analysis indicated that the high-expression groups had worse survival compared to the low-expression groups with the Kaplan–Meier method (Fig. 1c). This reinforced our hypothesis that lncRNA-NORAD might be an oncogenic gene that participated in the breast cancer progression.

### Inhibiting lncRNA-NORAD expression suppressed breast cancer cell proliferation, migration and invasion in vitro

**Fig. 2:**
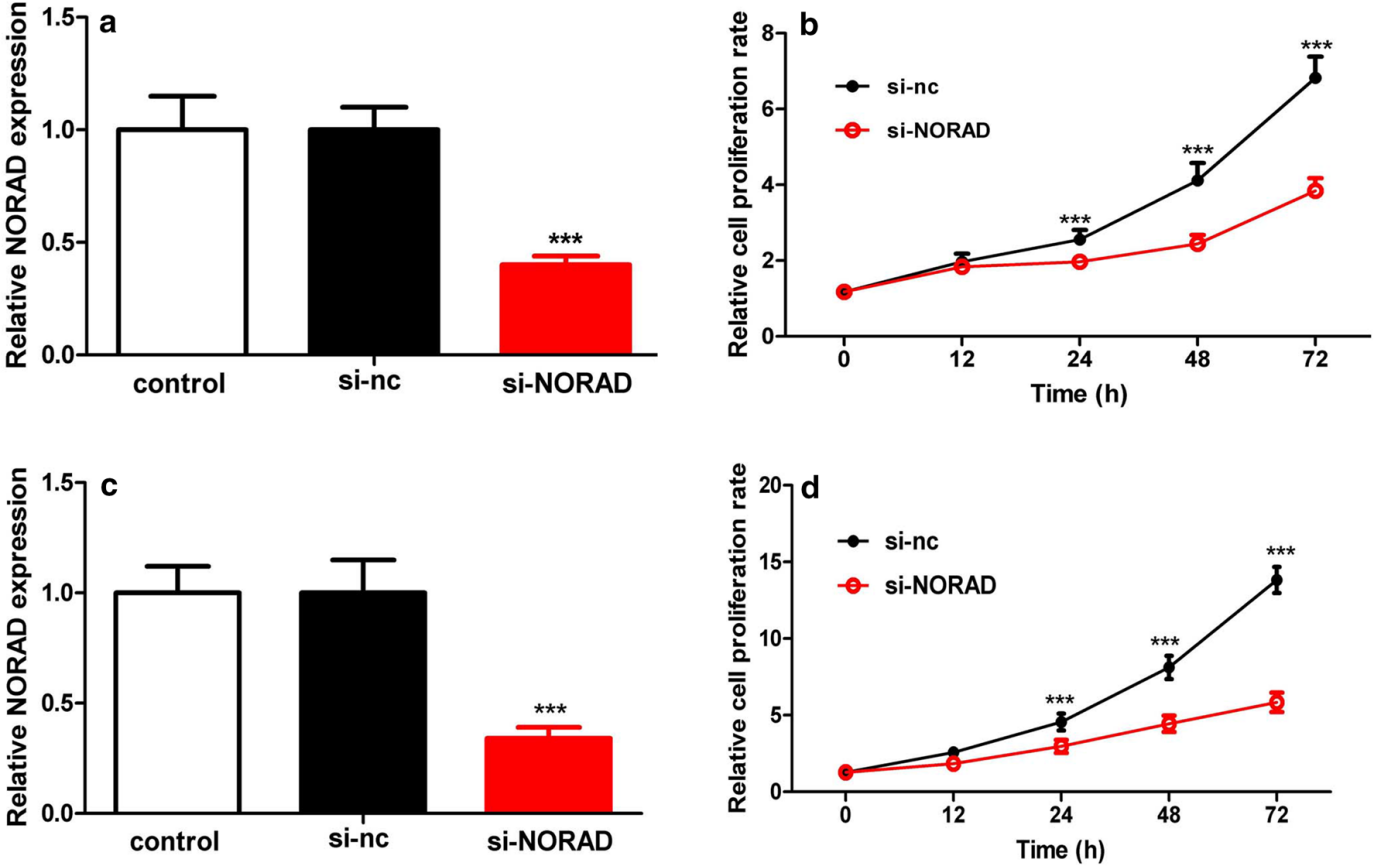
Linc-NORAD knockdown reduced cell migration and invasion. **a** Lnc-NORAD was knocked down by the si-NORAD interference RNA in MCF-7. Cells was transfected with a scrambled negative-control siRNA (si-nc) for comparison. **b** Cell proliferation rates were compared between the cells transfected with si-NORAD or si-nc. Lnc-NORAD knockdown by si-NORAD significantly inhibit MCF-7 breast cancer cells proliferation. **c** Comparison of proliferation of the MDA-MB-231 breast cancer cells transfected with si-NORAD or si-nc. **d** Si-NORAD significantly inhibit MDA-MB-231 cells proliferation. ***p < 0.001 vs the control

In order to investigate the influences of lncRNA-NORAD on cell proliferation, breast cancer cells MCF-7 and MDA-MB-231 were stably transfected with si-NORAD or si-nc. The expression level of lncRNA-NORAD in MCF-7 and MDA-MB-231 cells was significantly suppressed at least 50% of si-NORAD groups compared with that of negative control group si-nc (Fig. 2a, c). Meanwhile, the cell viability of MCF-7 and MDA-MB-231 cells, that determined by MTT assays, was significantly reduced in experiments (Fig. 2b, d). In conclusion, inhibiting lncRNA-NORAD expression could significantly inhibit breast cancer cell viability and proliferation.

**Fig. 3:**
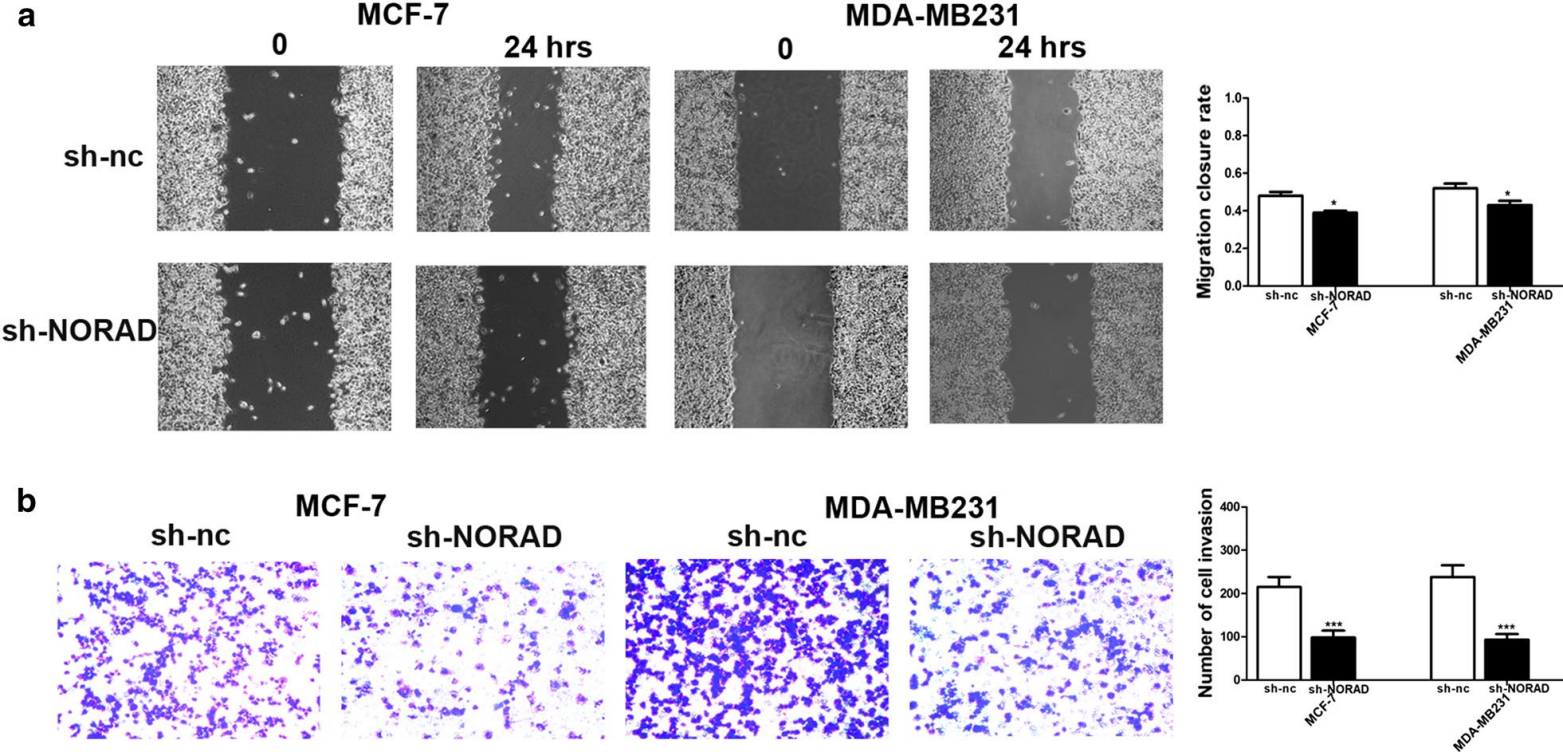
Lnc-NORAD knockdown reduced cell migration and invasion. **a** Comparison of migration of the MCF-7 and MDA-MB-231 breast cancer cells transfected with si-NORAD or si-nc. Si-NORAD led to migration inhibition in the MCF-7 and MDA-MB-231 cells. **b** Comparison of invasion of the MCF-7 and MDA-MB-231 breast cancer cells transfected with si-NORAD or si-nc. Si-NORAD led to invasion inhibition in the MCF-7 and MDA-MB-231 cells

We further try to investigate the influences of lncRNA-NORAD on cell migration and invasion in breast cancer cells. MCF-7 and MDA-MB-231 were stably transfected with si-NORAD or si-nc. Lnc-NORAD knockdown by si-NORAD led to both migration and invasion inhibition in the MCF-7 and MDA-MB-231 cells (Fig. 3a, b).

### Inhibiting lncRNA-NORAD suppressed the TGF-β signaling pathway in breast cancer cells

**Fig. 4:**
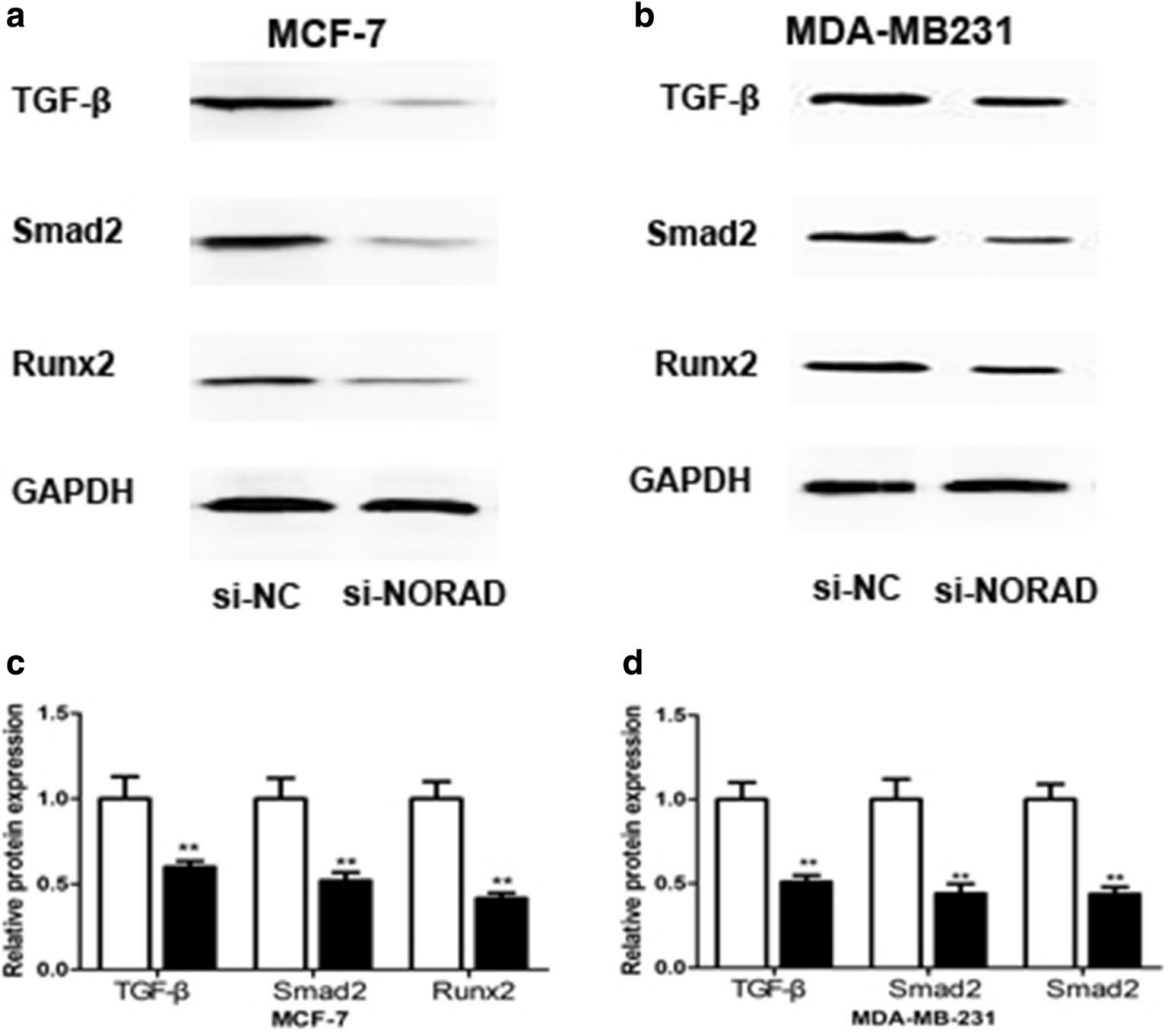
LncRNA-NORAD modulates the TGF-β signaling pathway. **a** The cells transfected with si-NORAD indicated TGF-β, Smad2 and Runx2 in the MCF-7 cells downregulated by western blot probing. The cells were transfected with si-NORAD or si-nc. **b** Quantitative comparison of the expression levels of TGF-β, Smad2 and Runx2 in MCF-7 cells. The protein expression levels in the MCF-7 cells transfected with si-nc were normalized to 1. **c**, **d** Comparison of the protein expression levels of TGF-β, Smad2 and Runx2 in the MDA-MB-231 cells. Data were mean ± SD derived from three independent experiments. **p < 0.01 vs the control

In order to uncover the mechanisms of lnc-NORAD promote the development of breast cancer, we analyzed lnc-NORAD impact on TGF-β by western blot. When lncRNA-NORAD was knocked down in the MCF-7 and MDA-MB-231 breast cancer cells, obviously down-regulated TGF-β expression levels were observed. And the downstream factors such as Smad2 and RUNX2 were also suppressed (Fig. 4a, b). These results robustly indicated that lncRNA-NORAD likely regulated the TGF-β signaling pathway and thus involved in the progression of breast cancer.

### LncRNA-NORAD knockout inhibited breast tumor growth in vivo

**Fig. 5:**
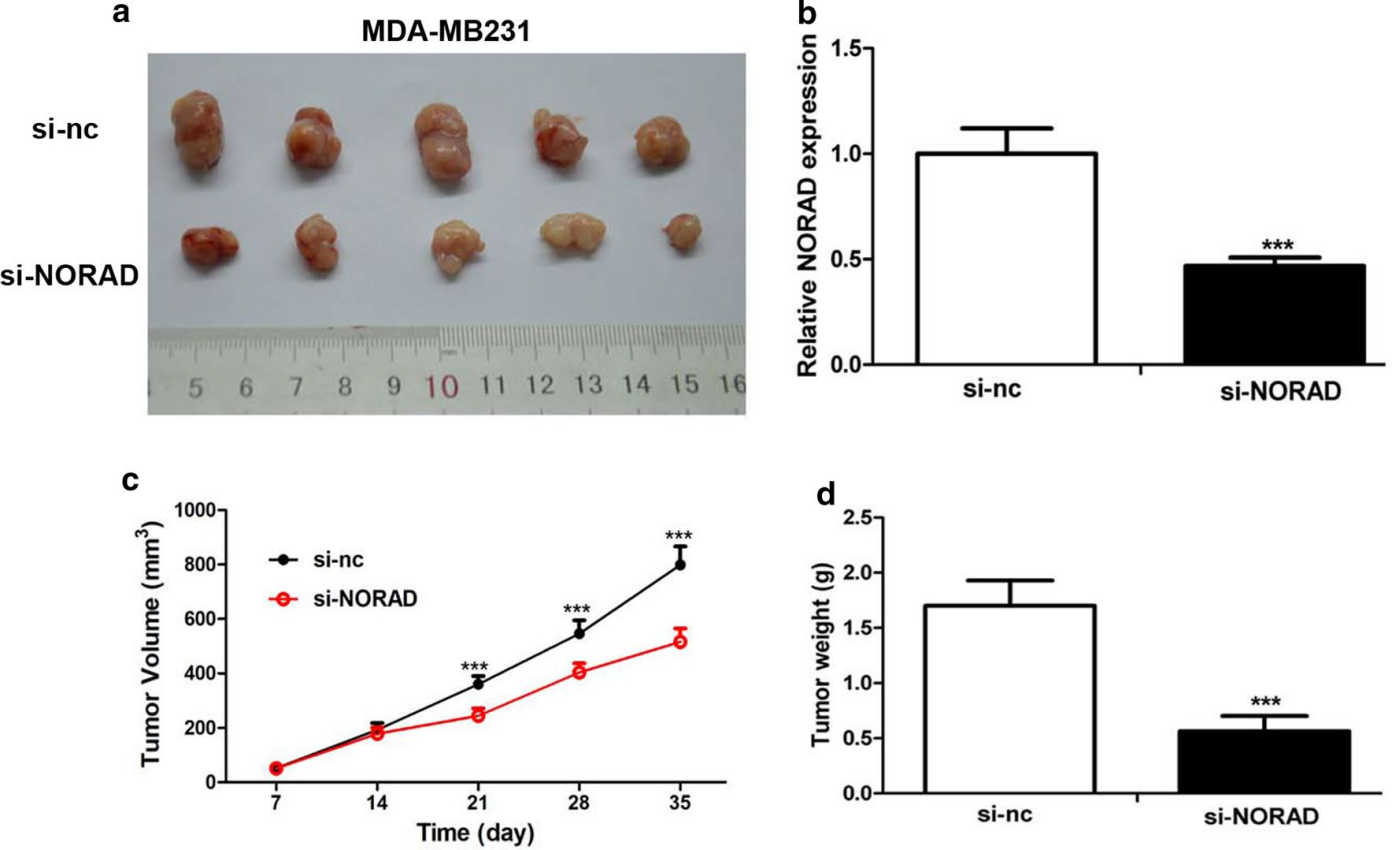
Lnc-NORAD knockdown reduced tumor growth in nude mice. **a** The MDA-MB231 cells were transfected with si-NORAD or si-nc and injected into nude mice. Tumor tissues were harvested and photographed 5 weeks later. **b** Relative lnc-NORAD expression was detected by RT-PCR in tumor tissues. The relative lnc-NORAD expression of tumors from the MDA-MB-231 cells transfected with si-NORAD or si-nc. **c** Time-course comparison of tumor growth from the MDA-MB-231 cells. Tumor sizes were measured once a week over 5 weeks and the tumor volume was calculated by L × S/2. **d** Comparison of tumors weight derived from the MDA-MB-231 cells, which were transfected with si-NORAD or si-nc and injected into nude mice. **p < 0.01 vs the control

We also compared the growth of tumors in the animals. The growth of the tumors formed from the injected cells was apparently smaller in the si-NORAD group during the period (Fig. 5a). And si-NORAD effectively suppressed the expression of lncRNA-NORAD in the MDA-MB231 transfected cells (Fig. 5b). In addition, the results of in vivo xenograft assays showed that tumor volume and weight of both si-NORAD groups were much smaller compared with those of si-nc group (Fig. 5c, d). These results suggested that inhibiting the expression of lncRNA-NORAD in breast cancer cells could efficiently suppress tumorigenesis in vivo. Through combining our above discoveries in cancer cells and tissue samples, we concluded that lncRNA-NORAD might be an important oncogene, and this lncRNA might function significant roles in cancer progression.

## Discussion

In the present study, high lnc-NORAD expression in both breast cancer cell lines and patient tumors was confirmed, and worse prognosis was observed in the high lnc-NORAD expression group. Our results have also demonstrated that lncRNA-NORAD regulated the TGF-β signaling pathway and might be responsible for promoting breast cancer progression. This is in line with TGF-β is a central signaling molecule in mammary tumorigenesis.

TGF-β is a kind of diversity and pleiotropic cytokines, with autocrine or paracrine manner through cell surface receptor signal transduction pathways to regulate the cell proliferation, differentiation, apoptosis, wound repair and immune function [11]. Many advanced tumors produce excessive amounts of TGF-β which, in normal epithelial cells, is a potent growth inhibitor [12]. However, in oncogenically activated cells, the homeostatic action of TGF-β is often diverted along alternative pathways. Hence, when carcinoma cells become refractory to TGF-β mediated growth inhibition, the tumor cell later in tumor development progresses by stimulating pathways with tumor effects [13]. Many studies have recognized that TGF-β participate in the malignant progression of breast cancer through promoting EMT [14, 15]. Our study has identified that overexpression of lnc-NORAD is constitutively related to the TGF-β signaling pathway in breast cancer. This suggests that the oncogenic activity of lnc-NORAD is at least through activating TGF-β signaling pathway for the progression of breast cancer in the advanced stages.

Runx2 is a lineage-specific transcription factor currently emerges as a key player involved in tumor metastasis [16, 17]. Recent studies also discovered Runx2 functioned an important role in promoting breast cancer metastasis [18–20]. Runx2 induced EMT evidenced by acquisition of a fibroblastic morphology, decreased expression of E-cadherin, increased expression of snail2 and vimentin. And TGF-β could upregulate Runx2 to promote breast cancer metastasis [21]. The present study has demonstrated that lncRNA-NORAD overexpression could increase the RUNX2 mRNA and protein expression levels, while knockdown lncRNA-NORAD inhibit the RUNX2 expression. Our results suggest that lncRNA-NORAD directly regulates the RUNX2 transcription. This might be that lncRNA-NORAD could regulate TGF-β signaling pathway and thus upregulate RUNX2.

## Conclusion

In summary, our experiments discover that lncRNA-NORAD is up-regulated in breast cancer tissues and cells, which promotes the breast cancer malignant progression via TGF-β signaling pathway and potentially related to RUNX2. Therefore, we identify that lncRNA-NORAD acts as an oncogenic RNA in the breast cancer tumorigenesis and the lnc-NORAD/TGF-β/RUNX2 axis in the breast cancer tumorigenesis.
